# Evolutionary trends in the emergence of skeletal cell types

**DOI:** 10.1093/evlett/qraf012

**Published:** 2025-05-18

**Authors:** Amor Damatac, Sara Koska, Kristian K Ullrich, Tomislav Domazet-Lošo, Alexander Klimovich, Markéta Kaucká

**Affiliations:** Max Planck Institute for Evolutionary Biology, Plön, Germany; Laboratory of Evolutionary Genetics, Division of Molecular Biology, Ruđer Bošković Institute, Zagreb, Croatia; Max Planck Institute for Evolutionary Biology, Plön, Germany; Laboratory of Evolutionary Genetics, Division of Molecular Biology, Ruđer Bošković Institute, Zagreb, Croatia; School of Medicine, Catholic University of Croatia, Zagreb, Croatia; Zoological Institute, Christian Albrecht University of Kiel, Kiel, Germany; Max Planck Institute for Evolutionary Biology, Plön, Germany

**Keywords:** cell type evolution, genomic phylostratigraphy, skeletogenesis, hypertrophic chondrocyte, lineage-specific genes

## Abstract

Cell types are fundamental functional units of multicellular organisms. The evolutionary emergence of new cell types is underpinned by genetic changes, such as gene co-option and cis-regulatory evolution, that propel the assembly or rewiring of molecular networks and give rise to new cell types with specialized functions. Here, we integrate genomic phylostratigraphy with single-cell transcriptomics to explore the evolutionary trends in the assembly of the skeletal cell type-specific gene expression programs. In particular, we investigate how the emergence of lineage-specific genes contributed to this process. We show that osteoblasts and hypertrophic chondrocytes (HC) express evolutionary younger transcriptomes compared to immature chondrocytes that resemble the ancestral skeletogenic program. We demonstrate that the recruitment of lineage-specific genes resulted in subsequent elaboration and individuation of the ancestral chondrogenic gene expression program, propelling the emergence of osteoblasts and HC. Notably, osteoblasts show significant enrichment of vertebrate-specific genes, while HC is enriched in gnathostome-specific genes. By identifying the functional properties of the recruited genes, coupled with the recently discovered fossil evidence, our study challenges the long-standing view on the evolution of vertebrate skeletal structures by suggesting that endochondral ossification and chondrocyte hypertrophy may have already evolved in the last common ancestors of gnathostomes.

## Introduction

During the evolutionary history of animals, a series of major evolutionary transitions were facilitated by the emergence of novel traits, which led to the enormous diversity and complexity of extant animals. The emergence of new cell types and their further individuation contributed to the origin of such functional and morphological novelties (Carter & Mess, 2007; Gans & Northcutt, 1983; Taylor & Padykula, 1978). Cell types are considered functional and evolutionary units that fuel innovations along the phylogeny of animals (Arendt et al., 2016a; Zeng, 2022). From an evolutionary standpoint, a cell type is a group of cells that share functional features and possess specific gene expression profiles that have arisen through the evolution of a particular regulatory program (Arendt et al., 2016b). Such regulatory programs are assembled through the integration of existing molecular modules, incorporation of novel genes, and evolution of the cis-regulatory landscape (Arendt et al., 2016b; Carroll, 2008; Wagner & Lynch, 2010). These mechanisms jointly contribute to the emergence of novel cell types and their further radiation. The cell types that evolved from the same ancestor usually share the expression of many genes (Arendt, 2005, 2008); therefore, the knowledge of the evolutionary origin of each gene is instrumental in understanding the origin, homology, and functional properties of a cell type.

The evolution of cell types has been conventionally studied by comparing the expression of a relatively small number of mostly conserved marker genes representing a cell type identity (Dufour et al., 2006; Meulemans & Bronner-Fraser, 2007; Tanabe et al., 1998; Tarazona et al., 2016). These investigations generated fundamental knowledge of cell type evolution and proposed homologies between numerous cell types. However, assessing such a limited set of genes does not provide a comprehensive overview of cell type evolution (Arendt, 2008), since the same gene can be a marker of several unrelated cell types. To complement the marker gene approach, alternative strategies identify core sets of co-regulated genes as molecular signatures of specific cell types (Tarashansky et al., 2021). Ultimately, because a cell's functional identity arises from the coordinated expression of thousands of genes, a whole-transcriptome approach can offer a more comprehensive framework for understanding cell type origins and functional differentiation. Here, we apply a genome-wide approach to explore the link between the evolution of protein-coding genes and the evolution of cell types through phylotranscriptomic analysis.

**Figure 1.**
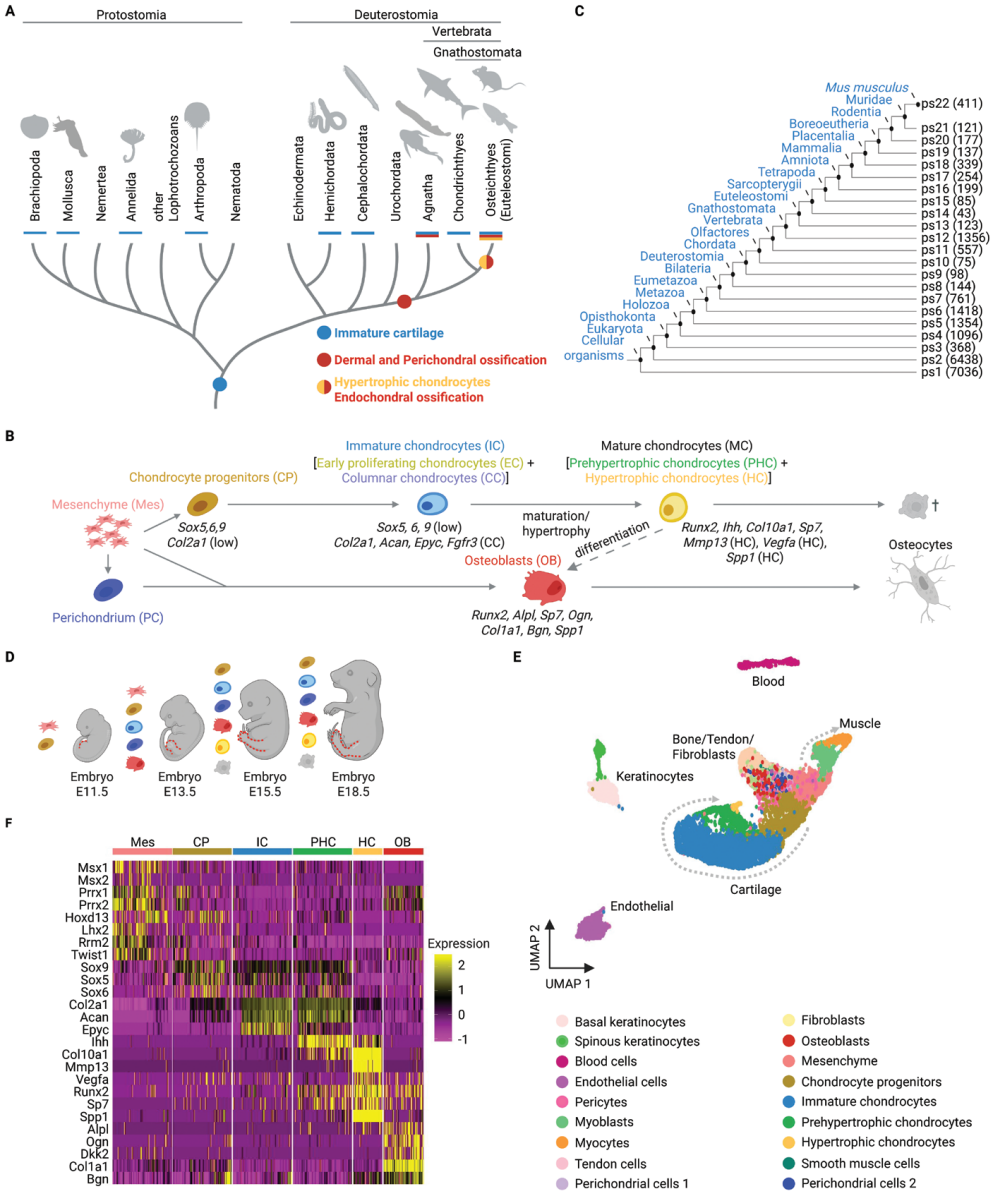
Paradigm and methodology to explore the contribution of lineage-restricted genes to the evolution of skeletal cell types. (A) The evolution of skeletal cell types in bilaterians. Colored dots indicate the current view on the evolutionary origin of cartilage (blue), dermal, perichondral, and endochondral bone (red). Chondrocyte hypertrophy (orange) is observed in Euteleostomi. Colored bars indicate the presence of skeletal tissues in a given lineage. (B) Schematic representation of cell types and their differentiation trajectories involved in the endochondral ossification during embryonic development. (C) Phylostratigraphy map of the mouse protein-coding genes. Each phylostratum (ps) number corresponds to a node in the phylogeny. Numbers in the parentheses indicate the number of genes originating in the phylostratum. (D) Embryonic stages covered by the single-cell transcriptome dataset from developing mouse hindlimb. Stage E11.5 contains mostly mesenchymal condensations; E13.5, E15.5, and E18.5 comprise cell types typically involved in chondrogenesis and osteogenesis in the limb. Mesenchymal cells condense within the developing limb bud and differentiate into chondrogenic and osteogenic cells. In the maturation process, chondrocytes undergo programmed cell death or contribute to the osteogenic lineage through transdifferentiation. (E, F) Single-cell transcriptome analysis. (E) The dataset was reanalyzed using the Seurat pipeline, and the cell clusters were projected using two-dimensional uniform manifold approximation and projection. Individual colors indicate the cell cluster identities. (F) Gene expression profile of skeletogenic cell clusters using cell type-specific genes.

The evolution of skeletal cell types had a major impact on the evolutionary novelties, adaptations, and transitions across animal lineages. In this study, we employed the skeletogenic lineage of the developing murine hindlimb as a model because it comprises diverse cell types that generate stiff elements in the process of endochondral ossification. In contrast to the formation of elastic and fibrous cartilage or dermal bones, where only a few cell types are involved, endochondral ossification is a bone-forming mechanism that employs the majority of skeletal cell types known in vertebrates, both from the chondrogenic and osteogenic lineages (Figure 1A). In vertebrates, chondrocytes produce cartilage that forms an embryonic blueprint of the future skeleton (Kronenberg, 2003). In most vertebrates, with the exception of cartilaginous fishes (Chondrichthyes), this template is subsequently substituted by mineralized bone. Chondrocytes that form cartilaginous supportive elements are also present in diverse invertebrate lineages (Figure 1A) (Cole & Hall, 2004; Gans & Northcutt, 1983; Person & Philpott, 1969), as was deducted from the shared expression of several marker genes and biochemical properties (Cole & Hall, 2004; Tarazona et al., 2016). This discontinuous presence of immature cartilage across the animal phylogeny, along with shared core genetic programs, suggests a common evolutionary origin of cartilage in the common ancestor of Bilateria (Figure 1A) (Brunet & Arendt, 2016; Tarazona et al., 2016).

While vertebrate immature cartilage is considered homologous to that found in nonvertebrate lineages (Gomez-Picos & Eames, 2015; Tarazona et al., 2016), hypertrophic chondrocytes (HC) and endochondral ossification are widely observed only in extant bony vertebrates—Euteleostomi/Osteichthyes (Figure 1A) (Cole & Hall, 2004; Donoghue & Sansom, 2002; Gillis, 2019; Gomez-Picos & Eames, 2015; Ryll et al., 2014). The first bones emerged in stem vertebrates, giving rise to dermal and perichondral bones (Figure 1A) (Donoghue & Sansom, 2002; Hall, 1975; Ryll et al., 2014; Zhu et al., 2013). This renders endochondral ossification a more evolutionary recent process compared to dermal and perichondral ossification. Chondrocytes and osteoblasts are known to share the expression of many genes (Abzhanov et al., 2007; Bonucci et al., 1992; Eames et al., 2012), suggesting a common evolutionary origin. Given the distribution and origin of cartilage and bone across phylogeny, as well as the evidence that the *Sox9* gene regulatory network (GRN) tends to be dominant over the *Runx2* GRN in skeletal cell fate decisions, it has been hypothesized that the osteoblast transcriptome evolved from the chondrogenic gene expression program (Fisher & Franz-Odendaal, 2012a; Gomez-Picos & Eames, 2015; Nguyen & Eames, 2020). However, compelling evidence for this hypothesis remains lacking, and the evolutionary dynamics driving the assembly and individuation of these transcriptomes are not yet fully understood.

In embryonic development, both chondrocytes and osteoblasts originate primarily from mesenchymal cells (Figure 1B). The chondrocyte differentiation trajectory spans from mesenchymal progenitors through proliferating immature chondrocytes (IC), to terminally differentiated mature chondrocytes (MC) (Eames et al., 2003, 2004). IC can be further subdivided into early chondrocytes (EC, also known as round or resting chondrocytes) and columnar chondrocytes (CC, also referred to as proliferating or flattened chondrocytes), while MC comprise prehypertrophic chondrocytes (PHC) and HC (Figure 1B) (Kronenberg, 2003; Nguyen & Eames, 2020). Each of these cell types is characterized by a specific combination of marker genes. The unique property of maturing chondrocytes in endochondral ossification is their ability to undergo a programmed cell death defined by distinct morphological and transcriptomic changes (Karsenty et al., 2009; Roach et al., 2004), referred here to as the hypertrophy. An alternative cell fate trajectory of MC is their differentiation into osteoblasts (OB) (Aghajanian & Mohan, 2018; Giovannone et al., 2019; Yang et al., 2014).

Here, we employ a comparative transcriptomic approach that combines genomic phylostratigraphy with single-cell transcriptomics to gain deeper insights into the evolution of skeletal cell types. We specifically ask how the evolution of lineage-specific genes contributed to the emergence of novel cell types and their biological function. We investigate the evolutionary origin of HC and show that the assembly of its genetic program coincides with the presence of the endochondral ossification in the ancestor of jawed vertebrates (Brazeau et al., 2020), suggesting the need to reconsider the current view on the skeletal cell type evolution.

## Results

To estimate the evolutionary age of genes, we employed genomic phylostratigraphy (Domazet-Loso et al., 2007, 2024). This method assigns a relative age to each gene according to the most distant phylogenetic node where sequence homology remains detectable (Domazet-Loso & Tautz, 2010b; Domazet-Loso et al., 2007). We carried out a phylostratigraphic analysis of all mouse protein-coding genes across 22 phylogenetic nodes (phylostrata, ps), starting from the common ancestor of cellular organisms to the terminal branch, *M. musculus* (Figure 1C; Supplementary Figure S1; Supplementary Tables S1–S3). The generated phylostratigraphic map indicates the evolutionary origin of 22,590 murine genes along the consensus phylogeny, and the obtained distribution is similar to the earlier observations (Neme & Tautz, 2013; Wilson et al., 2017). Previous studies utilized genomic phylostratigraphy to explore the evolution of developmental stages, organs, tissues, and cells acquired from heterogeneous samples that contained a mixture of different cell types (Domazet-Loso & Tautz, 2010a; Futo et al., 2021; Hemmrich et al., 2012; Quint et al., 2012; Sestak & Domazet-Loso, 2015; Sestak et al., 2013), demonstrating its capability to provide evolutionary insights into developmental processes. Therefore, combining information on gene origin with modern single-cell transcriptomics opens an opportunity to better understand the evolution of cell type-specific transcriptomes. Here, we applied genomic phylostratigraphy in the context of single-cell transcriptomics of the skeletal cell types.

To capture the gene expression program of skeletogenic cell types, we took advantage of a single-cell transcriptome dataset from murine hindlimb development (Figure 1D) (Kelly et al., 2020). We applied higher-resolution cell clustering compared to the original study and used specific skeletal markers to identify all relevant cell types, including IC, PHC, HC, and OB (Figure 1E and F; Supplementary Figure S2). The remaining clusters represent endothelial, myogenic, and hematopoietic lineages (Figure 1E; Supplementary Figure S2). We emphasize that the great advantage of the single-cell transcriptomes, compared to the bulk sequencing data, is that each cell type is represented by dozens or hundreds of individual cells, which serve as independent biological replicates. This enables a robust statistical comparison among cell types by leveraging the fact that each cell type is represented by multiple individual cell transcriptomes—a feature inherently lost in pooled bulk samples (Ma & Zheng, 2023). This aspect is essential for detecting evolutionary differences in skeletal cell types, allowing a deeper understanding of their molecular distinction.

**Figure 2.**
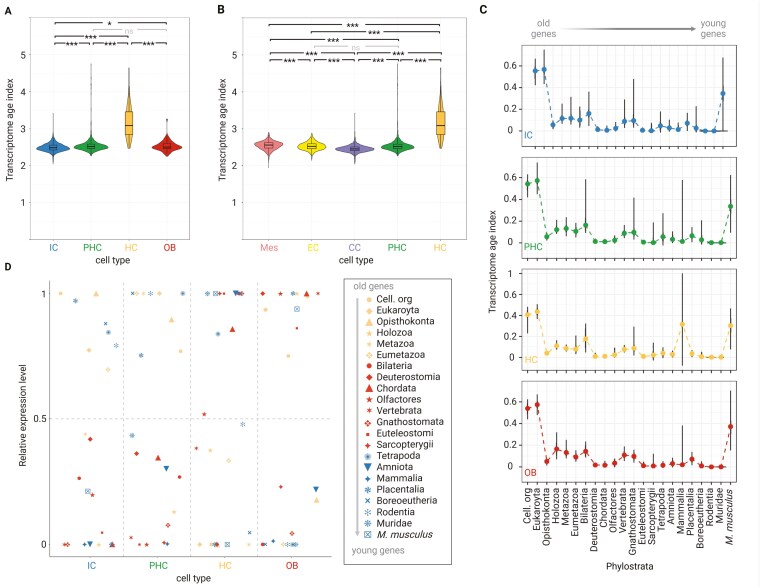
Phylotranscriptomic analysis of skeletal cell types. (A) Phylogenetic age of the skeletal cell types based on the transcriptome age index (TAI). A lower TAI value indicates a phylogenetically older transcriptome. All expressed genes in a given cell type are considered. Of note, the TAI calculation for OB was done irrespective of their cellular origin (whether OB differentiated from perichondrium, mesenchymal cells or HC is not distinguished) (B) Phylogenetic age of the cell types within the chondrogenic lineage, clustered in higher resolution to distinguish individual differentiation stages, based on the TAI. (C) Partial TAI (pTAI) of skeletal cell types split according to the origin of the genes from the different phylostrata. The violin plot in individual phylostrata shows the TAI values for every cell, while the line plot connects the median value in each phylostrata. (D) Relative expression of genes from different phylostrata across skeletal cell types. The mean expression of genes for a given ps in each cell type was linearly transformed to a relative expression interval (minimum expression = 0, maximum expression = 1). (A, B) The statistical significance of differences between TAI values was evaluated using a pairwise Wilcoxon test corrected for multiple comparisons by Benjamini and Hochberg (BH). Asterisks denote adjusted *p*-value levels (*p ≤ 0.05, **p ≤ 0.01, ***p ≤ 0.001). IC = immature chondrocytes; PHC = prehypertrophic chondrocytes; HC = hypertrophic chondrocytes; OB = osteoblasts; EC = early chondrocytes; CC = columnar chondrocytes.

To estimate the relative evolutionary age of the cell type-specific transcriptomes, we calculated the transcriptome age index (TAI) (Domazet-Loso & Tautz, 2010a) for each skeletogenic cell. The TAI integrates the average age of all protein-coding genes expressed in a cell with their expression level. A lower TAI value indicates an evolutionarily older transcriptome, while a higher TAI value corresponds to an evolutionarily younger transcriptome. We compared the TAI values of all cells among skeletogenic cell types (Figure 2A; Supplementary Figure S3).

First, we observe that chondrocytes (comprising both IC and MC) express, on average, older transcriptomes than OB (Supplementary Figure S3A). This is particularly evident when only the TAI of upregulated differentially expressed genes (DEGs) are compared between these cell types (Supplementary Figure S3A). This result agrees with the molecular, phylogenetic, and fossil evidence that cartilage has a more ancient evolutionary origin than bone (Brunet & Arendt, 2016; Cole & Hall, 2004; Mallatt & Chen, 2003; Rychel et al., 2006; Tarazona et al., 2016).

Because the chondrocyte population is heterogeneous and comprises both mature and immature states, we analyzed this population at a higher resolution and clustered the cells according to their maturity (ontogeny). The TAI of IC is lower than the TAI of MC (comprising PHC and HC) and osteoblasts (OB) (Figure 2A). Interestingly, HC demonstrate substantially higher TAI values than OB and all other chondrocyte cell types. These patterns remain consistent when using only specifically upregulated genes or when randomly downsampling the number of cells for each cell type (Supplementary Figure S3B–D), indicating the robustness of TAI patterns. As the TAI calculation incorporates gene expression level as a weight factor (Domazet-Loso & Tautz, 2010a; Ma & Zheng, 2023), these results indicate that the higher TAI of OB and HC are influenced by the stronger influence of recently evolved genes as compared to IC. Most notably, our findings suggest that HC possess the youngest transcriptome among the skeletal cell types, even younger than OB. By partitioning the immature chondrocyte population even further, we observe that CC, representing the last immature chondrogenic state, express a transcriptome that is older than any other tested cell type (Figure 2B). This suggests that CC transcriptome might represent the oldest chondrogenic (or skeletogenic) genetic program among the skeletal cell types.

To reveal the contribution of the individual phylostrata to the TAI value, we calculated the partial TAI (pTAI) scores according to the origin of genes from the different phylostrata (Domazet-Loso & Tautz, 2010a) (Figure 2C; Supplementary Figure S4). To simplify the presentation of these results, we connected the median of partial TAI values in each phylostrata for each skeletal cell type (Figure 2C). We observe that genes originating from cellular organisms (ps1) to Bilateria (ps7) contribute greatly to the global TAI values. Notably, these ancient genes include *Sox*, *Runx*, and collagen gene families that are conventionally used as marker genes for the skeletal cell types (Supplementary Table S1). This observation is consistent with the previous findings that the marker genes of skeletal cell types consist of ancient genes that have been co-opted into their GRNs during evolution (Fisher & Franz-Odendaal, 2012b; Zhang et al., 2006). Consequently, this observation supports that the ancient core genes involved in the first skeletal (chondrogenic) gene expression program were assembled during the emergence of Bilateria and have been conserved, as evidenced by the existence of cartilage in several protostome and deuterostome lineages (Cole & Hall, 2004; Tarazona et al., 2016). Comparison of pTAI values among skeletal cell types (Supplementary Figure S4) reveals that most ancient genes (cellular organisms- Opisthokonta) are more expressed by IC, further suggesting that IC is most reminiscent of the ancient chondrogenic genetic program.

We find that genes that have emerged later in the evolution, including genes from Vertebrata (ps11), Gnathostomata (ps12), and *M. musculus* (ps22), also contribute substantially to the global TAI score (Figure 2C). Particularly, OB show a significantly high pTAI at the onset of Vertebrata (ps11), while HC exhibit considerably elevated pTAI in Gnathostomata (ps12) (Supplementary Figure S4). These observations suggest that the corresponding phylogenetic nodes were important periods in the evolution of OB and HC, respectively, and contributing to the transcriptome divergence from the ancient skeletogenic genetic program. We also detect a strong surge of pTAI in HC at the origin of Mammalia (ps17), where solely a high expression of *Spp1* (Osteopontin), a marker of HC, causes the substantial pTAI increase. This indicates that *Spp1* is an important component of the HC gene expression program, and its high expression is required for mineralization and bone properties (Foster et al., 2018).

To better understand the individuation of skeletal cell type transcriptomes, we compared the proportions of shared and uniquely expressed genes among IC, HC, and OB along the ps nodes (Supplementary Figure S5). Generally, IC, OB, and HC share a large proportion of genes, in line with their proposed shared evolutionary origin (Gomez-Picos & Eames, 2015). We note that from all pairwise comparisons across ps, OB and HC exhibit the least proportion of shared genes, while each of them shares more genes with IC, suggesting their independent evolution from the ancestral skeletal gene expression program. We performed a Gene Ontology (GO) enrichment analysis of the shared and unique genes to identify the different biological functions associated with these gene sets (Supplementary Figure S6; Supplementary Tables S4–S9). We find that genes unique to IC are associated with the known properties of cartilage, such as cartilage development and synthesis of proteoglycan and aminoglycan. HC and IC share genes associated with cartilage development, while OB and IC share genes related to ECM organization. Genes unique to OB and HC are associated with their distinct biological properties, including ECM organization, ossification, limb and skeletal development for OB, and cell death, ossification, cartilage, and bone development, and cytoskeleton organization for HC. Given that IC likely represent the ancestral cell type (Fisher & Franz-Odendaal, 2012a; Tarazona et al., 2016), these findings suggest that HC and OB transcriptomes may have evolved from a common ancestral (chondrogenic, IC-like) gene expression program. In summary, the smaller gene overlap between OB and HC, their unique functional properties, and their differing phylogenetic distributions support the hypothesis that HC and OB may have evolved independently. Overall, our observations from TAI and Venn diagram analyses suggest further elaboration of the ancestral skeletogenic gene expression toolkit, whereby the acquisition of lineage-specific genes may facilitate transcriptome individuation.

To further demonstrate to what extent genes of particular evolutionary origin contribute to the overall cell type-specific transcriptomic profile, we performed a relative expression analysis of genes evolved in different phylostrata (Figure 2D). We show that IC and PHC highly express mainly ancient genes that originated between the origin of cellular organisms and the radiation of Eumetazoa (ps1-6, orange shapes). In contrast, evolutionarily younger genes that emerged in Bilateria (ps7) or later (red and blue shapes) are more expressed by either OB or HC. Comparisons of the average expression of genes from each ps and among cell types provide statistical support that older genes tend to be more expressed by IC, while younger genes are more expressed in OB and HC (Supplementary Figure S7). Taken together, the analysis of the shared and unique gene composition (Supplementary Figures S5 and S6) and the relative expression of the genes originating in different phylostrata (Figure 2D; Supplementary Figure S7) highlights the contribution of evolutionarily younger genes to individuation of the OB and HC gene expression programs and supports their more recent evolutionary origin compared to IC.

**Figure 3.**
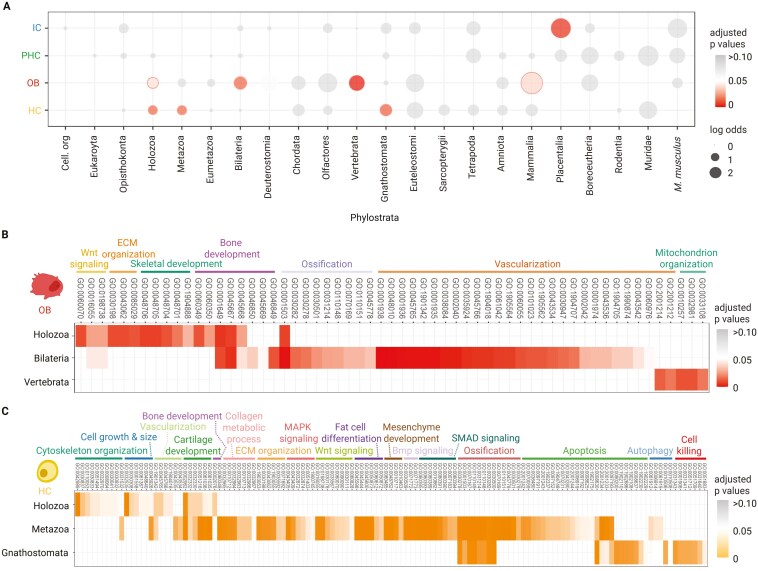
Functional enrichment of genes associated with the evolution of skeletal cell types. (A) Enrichment analyses on upregulated DEG distribution across the phylogeny. The representation of novel genes associated with the evolution of a particular cell type is shown for each ps in log-odds values. Circle colors indicate the statistical significance of gene enrichment associated with the evolution of a particular cell type. Enrichments were tested using a two-tailed hypergeometric test corrected for multiple comparisons by FDR (*p* ≤ 0.05). (B, C) Gene ontology (GO) enrichment analysis for biological processes of differentially expressed genes originated from the significantly enriched phylostrata in (A). All enrichment analyses were corrected by BH (*p* ≤ 0.05). Enriched phylostrata, including Placentalia (IC) and Mammalia (OB), did not provide significant GO terms. A complete list of statistically significant GO terms is provided in the Supplementary Tables.

TAI is a measure that considers all gene expression levels via their partial concentrations (Domazet-Loso & Tautz, 2010a). However, transcriptome data could also be assessed by looking for statistical enrichment of all upregulated transcripts regardless of their partial concentrations in a cell. This type of analysis gives equal weight to highly expressed genes and those represented by a small number of transcripts (Corak et al., 2023). We thus performed enrichment analysis on all upregulated genes of skeletal cell type transcriptomes (Figure 3A). We observe significant enrichment of genes in several phylostrata such as Holozoa (ps4; for both OB and HC), Metazoa (ps5; for HC), Bilateria (ps7; for OB), Mammalia (ps17; for OB), and Placentalia (ps18; for IC). Interestingly, a significant number of recently evolved genes that are part of the OB and HC transcriptomes appeared at the onset of Vertebrata (ps11) and Gnathostomata (ps12), respectively, which aligns well with patterns previously deducted from pTAI and relative expression analysis. These results further show that genes originating in ps11 and ps12 have been recruited specifically into the OB and HC gene expression programs. While our analysis cannot demonstrate whether these genes were incorporated into the gene expression programs at the time of the genes’ origin, it indicates the earliest point when such integration was possible. Our findings suggest that the genes from the enriched phylostrata, particularly genes of more recent origin, are involved in the adaptive history of skeletal cell types. For OB, the enrichment in ps11 coincides with the reported first appearence of bones in vertebrates (Donoghue & Sansom, 2002; Hall, 1975). In the case of HC, the significant enrichment of genes in ps12, the indispensability of HC for endochondral ossification and a recent fossil finding of extensive endochondral bone in Early Devonian placoderm-like fish *Minjinia turgenensis*, stem-group gnathostome (Brazeau et al., 2020), jointly suggest that HC emerged at the onset of jawed vertebrates (Gnathostomata), instead of bony vertebrates (Osteichthyes) as previously thought (Donoghue et al., 2006).

To better understand the molecular and functional modules associated with these phylostratum enrichments in HC and OB, we performed GO enrichment analysis on the genes from each enriched phylostratum in Figure 3A. (Figure 3B and C; Supplementary Tables S10–S15). For OB, the genes originating in older phylostrata (Holozoa and Bilateria) are associated with *Wnt* signaling, extracellular matrix (ECM) organization, bone development, ossification, and vasculogenesis. In Holozoa, these include known OB transcription factors from the *Runx* and *Twist* gene families. In Bilateria, we recovered enrichments from ossification- and vasculogenesis-related genes. OB furthermore utilize genes related to mitochondrion organization that have emerged in Vertebrata (ps11), which may be required for the specific energetic and metabolic demand during the mineralization process (Boonrungsiman et al., 2012). Notably, OB also employ novel vasculogenesis-related genes *Ramp2* and *Tmem100* from the node Vertebrata (ps11) that enabled the recruitment of the preexisting vasculogenesis module into the new context of bone formation (Ichikawa-Shindo et al., 2008; Somekawa et al., 2012). Vascularization, a hallmark feature of bone formation, is considered a major evolutionary innovation that distinguishes bone from the ancestral skeletal tissue, the nonvascularized cartilage (Filipowska et al., 2017; Sivaraj & Adams, 2016). Overall, these newly evolved components of the transcriptome and their associated functions align well with the described biology of bone formation.

In Holozoa (ps4), HC-associated genes were assigned to GO terms linked with cytoskeleton organization, cell growth, and size, which may be related to the ability of chondrogenic cells to change their shape and increase in size—a hallmark feature of HC during the chondrocyte maturation process (Farnum et al., 2002; Woods et al., 2007). Holozoan genes are additionally associated with GO terms related to cartilage and bone development. Noteworthy among them are key regulator genes like *Runx* and *Smad* family members, recognized for their essential roles in the development of mature cartilage and bone (Alvarez & Serra, 2004; Komori, 2005; Qin et al., 2020). HC-associated genes of metazoan origin (ps5) are linked to key signaling pathways such as *Wnt*, MAPK, Bmp, and SMAD that are known to facilitate chondrocyte differentiation and maturation (Chen et al., 2021). HC also utilize holozoan and metazoan genes related to bone development and ossification, which align well with the HC’s involvement in endochondral ossification. Ancient metazoan genes that are part of the HC transcriptome, including *Bax*, *Cflar*, *Mcl1*, *Lgals3*, *Wfs1*, *Pabpn1*, *Nr4a2*, and *Wnt4*, are linked to the apoptotic pathway (Beard et al., 2015; Fulda, 2009; Gong et al., 2021; Harazono et al., 2014; Oltvai et al., 1993; Sasseville et al., 2006; Wei et al., 2015). Notably, the HC transcriptome is further complemented by genes of Gnathostomata (ps12) origin that are known to modulate programmed cell death, such as *Bid*, *Bcl2l1*, *Bmf*, *Ier3*, and *Muc1* (Arlt & Schäfer, 2011; Giam et al., 2008; Korsmeyer et al., 2000; Opferman & Kothari, 2018; Supruniuk & Radziejewska, 2021; Wei et al., 2001). These observations provide the first molecular evidence that the evolution of HC was fueled by the acquisition of cell death control, a hallmark process of HC during hypertrophy (Aghajanian & Mohan, 2018; Karsenty et al., 2009). In summary, our findings indicate that the evolution of skeletal cell types (OB and HC) follows a general principle of co-opting key regulatory genes and acquiring functional modules (*i.e*., vasculogenesis and cell death), with significant contributions from evolutionarily younger novel genes. In particular, the analyses additionally pinpointed specific vertebrate- and gnathostome-specific genes that provided evolutionary younger skeletal cell types, OB and HC, with the ability to modulate ancient functions.

To test whether the evolutionary trends of skeletal cell type emergence observed in the mouse dataset can be recapitulated in another vertebrate species, we complemented our study using zebrafish (*Danio rerio*). This is another widely used vertebrate model, representing a basal group in Osteichthyes, with available single-cell transcriptome data comprising skeletal cell types. To capture all skeletal cell types, we repeated the phylotranscriptomic analysis using two zebrafish developmental datasets (Supplementary Figures S8 and S9; Supplementary Tables S16–S18). However, since the zebrafish skeleton is predominantly formed by intramembranous ossification (Le Pabic et al., 2022), capturing HC proved challenging. Using the skeletal marker genes from the mouse dataset, we identified IC and OB in the first dataset (Fabian et al., 2022) at the stages 5 days postfertilization (5 dpf) and 14 days postfertilization (14 dpf) (Supplementary Figure S8B). In the second dataset (Lange et al., 2024), IC is also present at 5 dpf; however, we observed that the *col10a1a*-positive cell population expresses a mixed signature of both HC (*ihha*-positive) and OB (*col1a1a*-positive). Therefore, we assign these cells as an HC/OB cluster expressing both *ihha* and *col10a1* for the following analyses (Supplementary Figure S8B). In the TAI analysis, both OB and HC/OB exhibit higher TAI than IC, consistent with their more recent evolutionary origin compared to IC (Supplementary Figure S8C). Phylostrata enrichment analysis revealed that the zebrafish OB transcriptome is significantly associated with genes originating in Opisthokonta, and, similarly to mouse OB, in Holozoa (Supplementary Figure S8D). GO enrichment analyses of genes from these enriched phylostrata revealed that OB-related genes are linked to biological processes involved in bone biology, including ECM organization, skeletal development, protein kinase/transferase activity, and phosphorus/phosphate metabolism (Supplementary Figure S8E). Notably, we identified key transcription factors from the *Runx* family, in line with biological processes associated with OB in mouse. For the HC/OB population, we detected enrichment signals in Holozoa, Metazoa, Bilateria, Vertebrata, and Gnathostomata (Supplementary Figure S3D), mirroring the enrichment patterns observed in mouse HC and OB. GO enrichment profiling of genes from these phylostrata revealed their involvement in skeletogenesis-related biological processes. Particularly, as in mouse HC, zebrafish HC/OB include transcription factors from the *Runx* family and genes related to apoptosis (Supplementary Figure S8F). Overall, the analyses performed using zebrafish datasets corroborate our results in mouse and provide further support to the emergence of skeletal cell types through the step-wise recruitment of transcription factors and lineage-specific novel genes, enabling specialized functions.

## Discussion

Cell type emergence is a complex evolutionary process driven simultaneously by co-option, novel gene acquisition, and the evolution of regulatory noncoding elements (Arendt, 2020; Babonis et al., 2022; Carroll, 2008; Jenike et al., 2023; Kin et al., 2015, 2022; Londin et al., 2015; Nowakowski et al., 2018). Here, we set out to understand the contribution of different phylogenetic levels via protein-coding genes to the evolution of skeletal cell types. The evolution of skeletal tissues has been studied for decades, allowing us to compare our phylotranscriptomic findings with the extensive evidence from paleontology, phylogenetics, and molecular research. Current knowledge of the skeletal cell types evolution is mainly derived from the comparative biochemical, histological, and marker gene expression surveys. Previous investigations have assessed the expression of a limited number of selected marker genes from the *Sox, Runt*, hedgehog, and collagen gene families to deduce the cell type origin (Han & Lefebvre, 2008; Hecht et al., 2008; Jandzik et al., 2015; Meulemans & Bronner-Fraser, 2007; Rychel et al., 2006; Tarazona et al., 2016). However, these gene families are evolutionarily ancient and might be expressed by multiple unrelated cell types (Barrionuevo & Scherer, 2010; Lauri et al., 2014; Suzuki et al., 2006). While these studies uncovered numerous commonalities among skeletal tissues in both vertebrate and invertebrate lineages (Figure 1A) (Brunet & Arendt, 2016; Tarazona et al., 2016), they raised questions about the mechanisms fueling the skeletal cell types evolution, whether the skeletal cell types evolved from a common ancestral cell type, or how their genetic programs diverged. Here, we demonstrate the remarkable power of single-cell phylotranscriptomic analysis in addressing such inquiries, focusing specifically on how the evolution of lineage-restricted genes contributed to the emergence of novel cell types and their functional properties. We explored the evolutionary history of the gene expression programs operating in skeletal cell types and pinpointed the essential role of novel genes in this evolutionary process.

**Figure 4.**
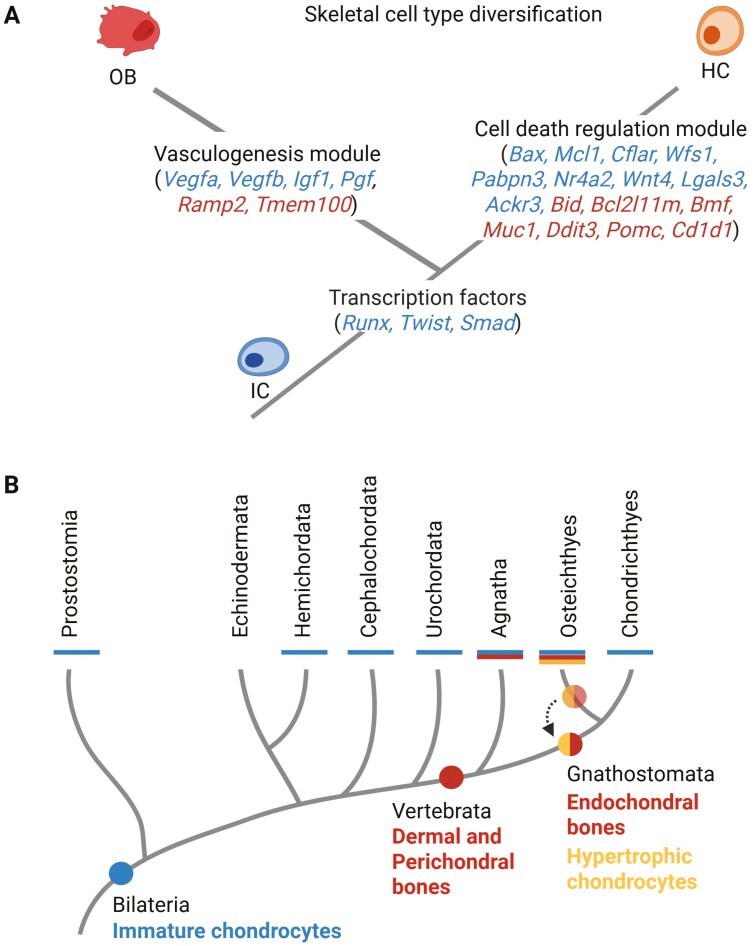
The molecular evolution and origin of skeletal cell types. (A) Skeletal cell types diversification occurred through the recruitment of transcription factors and the integration of additional functional modules. We found that the implementation of genes modulating vasculogenesis and mitochondrion organization in OB and programmed cell death in HC contributed to the individuation of the ancestral IC expression program. (B) Simplified phylogenetic origin of skeletal cell types in animals. We suggest that endochondral ossification and HC evolved in the common ancestor of Chondrichthyes and Osteichthyes, moving the origin of HC from the ancestors of bony vertebrates.

We demonstrate the use of TAI in inferring the evolutionary age of skeletal cell types transcriptomes. Using transcriptome age to explore the evolution of cell types relies on the notion that cell types expressing older transcriptomes (having lower TAI) are likely more ancient and tend to be functionally conserved (Ma & Zheng, 2023). Given this assumption, IC expressing the oldest transcriptome likely represent the prototypical skeletal (chondrogenic) expression program conserved among bilaterians. This finding is corroborated by the similarities of the immature cartilage across protostome and deuterostome lineages in the structural and biochemical properties and expression of core marker genes (Tarazona et al., 2016). Our analysis is consistent with OB and HC emerging later in the phylogeny and suggests an independent divergence and individuation from the ancestral gene expression program (Figure 4). Notably, HC express a younger transcriptome compared to OB, which supports the observed distribution of bones and mature cartilage on the vertebrate phylogeny (Figure 1A). However, we also found that the HC gene expression program may have evolved earlier than previously thought. Specifically, the pTAI, relative expression, and phylostratum enrichment analyses jointly point to the significant contribution of gnathostome-specific genes to the HC transcriptome, which raises the possibility that the last common ancestor of gnathostomes may have already possessed a complete HC gene expression toolkit. Since chondrocyte hypertrophy is exclusively involved in endochondral ossification (Aghajanian & Mohan, 2018; Kronenberg, 2003), it seems plausible that both HC and endochondral ossification emerged together. A recent paleontological study (Brazeau et al., 2020) identified the presence of endochondral bone in a placoderm fish, an ancestor of living gnathostomes. Therefore, the fossil evidence, together with the observed enrichment of gnathostome genes in HC, suggests that HC and endochondral ossification may be a gnathostome-specific novelty (Figure 4B). While the molecular and paleontological evidence is promising, further investigation is required to prove this hypothesis. However, these complementary findings offer an opportunity to reconsider the origin of HC and endochondral ossification in the ancestor of gnathostomes. Consequently, this evidence supports the emerging assertion that Chondrichthyes have lost the ability to make all types of bones secondarily (Brazeau et al., 2020;  Vaškaninová, 2020), while also raising the question of whether an HC-like program existed in stem Chondrichthyes.

Examining the entire cell type-specific transcriptomes allowed us to shed light on their evolutionary step-wise assembly and uncover the contribution of the ancient and novel genes to the emergence and individualization of skeletal cell types’ gene expression programs. Here, we identify genes that originated from particular evolutionary nodes that provided the cell types with new distinct biological identities and functions. This facilitates the individuation from the ancestral chondrogenic genetic program and drives the emergence of the evolutionarily younger cell types, OB and HC (Figure 4A). We show that the recruitment of transcription factors from Holozoa (i.e., *Runx, Twist, Smad*) contributed to the cell type diversification from the ancestral chondrogenic cell type, supporting the proposed principle that the evolution of cell types requires changes in transcription factors to create new cell type identities. In conjunction, the employment of lineage-specific genes enables the integration and modulation of ancient processes (preexisting biological functions) as new cellular modules of HC and OB (Figure 4A). The recruitment of genes controlling the ancient programmed cell death machinery facilitated the evolution of HC, while genes modulating vasculogenesis propelled the emergence of OB. As such, these findings propose these skeletal cell types to be examples of evolutionary terminal additions that result in modifications in cell–cell signaling and the creation of novel cellular niches (Arendt, 2008; Torday & Miller, 2018). Our findings complement well the observations of the role of signaling pathways innovations in the evolution of cell types (Wagner et al., 2019) and support the emerging view that the evolutionary origin of many novel cell types lies in the GRNs behind the response of a cell to its environment.

While the functional role of individual genes is yet to be experimentally validated, these findings support the notion that cell type evolution is linked to the acquisition of new functions (Callier, 2020). Interestingly, the origin of some of the lineage-restricted genes that comprise the cell type-specific transcriptome coincides with the emergence of the respective cell types (vertebrate-specific genes in OB; gnathostome-specific genes in HC), providing a mechanistic link between the evolution of novel genes, the acquisition of new cellular functions and cell type emergence. While our applied research strategy cannot reveal whether the novel genes were incorporated into the cell type-specific gene expression program at the time of the genes’ origin, it clearly pinpoints the very first moment when such integration was possible. In the future, comparative single-cell phylotranscriptomic analyses of corresponding skeletal tissues across the Tree of Life will clarify the time of the genes’ integration.

In summary, we present a study that may inspire the design principles for future investigations tailored specifically to questions of a particular cell type evolution and individuation of gene expression programs from their ancestor. The recent advances in single-cell omics technologies deliver complex and high-resolution information on the molecular profile of individual cells. Examining several thousands of genes in each cell provides a comprehensive insight into the cell identity along a developmental trajectory (Tang et al., 2009). The implementation of additional tools for inferring gene homologies, such as BLAST-independent HMM approaches (for instance, see (Anari et al., 2018; Barrera-Redondo et al., 2023; Rossier et al., 2021)), incorporation of an ever-increasing knowledge of novel gene emergence, and methodologies to distinguish between duplication-divergence and de novo evolution, the synteny analysis, will promote a broader and even more confident application of genomic phylostratigraphy on single-cell data to address questions of cell type evolution.

## Methods

### Single-cell transcriptome datasets

We employed published 10x Genomics single-cell transcriptome datasets from mouse and zebrafish. In the mouse dataset, samples of the developing hindlimb were recovered from four embryonic stages (E11.5, E13.5, E15.5, and E18.5) (Kelly et al., 2020). Additionally, we utilized two zebrafish datasets: one covering the larval stages 5 dpf and 14 dpf and comprising cell progenies that emerged from labeled neural crest cells in the head region (Fabian et al., 2022), and the second dataset analyzing larval stage 5 dpf and a whole embryo (Lange et al., 2024).

### Single-cell transcriptome analysis

In the single-cell dataset of mouse limb development, the raw count matrix was processed using the Seurat v3 (Stuart et al., 2019) R package. We filtered out cells with less than 200 and more than 5,000 genes. Cells with more than 20% mitochondrial read content were also removed. The genes with either zero expression in all cells or only expressed in less than ten cells were excluded. The filtered data were normalized and variance stabilized (cell cycle phase and mitochondrial percentage regression) using the SCTransform algorithm (Hafemeister & Satija, 2019). The stages were then integrated using the highly variable genes identified using SCTransform. To visualize the integrated data, the resulting file was subjected to dimensionality reduction methods such as Principal Component Analysis and Uniform Manifold Approximation and Projection. We clustered the cells using Seurat’s default settings (*K*-nearest neighbor (KNN) graph and Louvain algorithms). After processing, we retained 9,599 cells with a total of 17,253 genes expressed. The cell clusters were identified using the upregulated DEGs obtained using Seurat’s *FindMarkers* command and known cell type-specific markers found in the literature.

### Genomic phylostratigraphy mapping of protein-coding genes in *Mus musculus* and *Danio rerio* genomes

The phylostratigraphic procedure was performed as previously described (Domazet-Loso et al., 2007; Domazet-Loso & Tautz, 2010a, 2010b). Consensus phylogenies covering divergence from the last common ancestor of cellular organisms to *M. musculus* and *D. rerio* as focal organisms were constructed following the recent phylogenetic literature (D’Elia et al., 2019; Delsuc et al., 2008; Harvey et al., 2021; Hughes et al., 2018; Steppan & Schenk, 2017; Swanson et al., 2019; Upham et al., 2019; Zou et al., 2012). The nodes were chosen based on their phylogenetic support from the literature, the availability of reference genomes for terminal taxa, and their importance in major evolutionary transitions, such as the emergence of major clades that mark innovations in body plan and function (e.g., Bilateria, Deuterostomia, Vertebrata, and Gnathostomes). The complete set of protein sequences for 513 (mouse) and 506 (zebrafish) terminal taxa was retrieved from Ensembl and NCBI databases. The protein sequence database was prepared for sequence similarity searches by checking the consistency of the files, leaving only the longest splicing variant per eukaryotic gene and adding taxon tags to sequence headers. The phylostratigraphy map of *M. musculus* and *D. rerio* was constructed by comparing 22,769 *M. musculus* and 25,787 *D.rerio* protein sequences with the protein sequence database by *blastp* algorithm V2.9.0 with a 10^−3^ e-value threshold (Altschul et al., 1990). Protein sequences that did not return their sequence as a match were discarded, which left 22,590 and 25,721 protein sequences in mouse and zebrafish, respectively. The mouse and zebrafish protein sequences were mapped on consensus phylogenies with 22 and 16 phylostrata (ps), respectively. Each protein was assigned to the oldest internode on the phylogeny, where it still had a match (Domazet-Loso et al., 2007; Domazet-Loso et al., 2024, Loso and Tautz 2010b,a).

### Transcriptome age index

We generated a normalized relative count matrix from the Seurat object using the total count normalization method multiplied by a scaling factor of 10^6^. The metadata was extracted from the Seurat file to provide the identities of each cell in the count matrix. The gene expression matrix was combined with the phylogenetic age assignment of genes from the phylostratigraphy map to calculate each cell’s TAI (Domazet-Loso & Tautz, 2010a). TAI was calculated using myTAI (Drost et al., 2018) R package based on the formula:


$$\begin{array}{l} TAI=\frac{\overset{n}{\underset{i=1}{\sum }}ps_{i}e_{ic}}{\overset{n}{\underset{i=1}{\sum }}e_{ic}} \end{array}$$

where the $ps_{i}$ represents the phylostratum assignment of gene i and $e_{ic}$ denotes the normalized read count of gene i in a cell (c), and n is the total number of expressed genes in a cell (c). We then projected the TAI values of each cell according to their cell type identities to infer the relative age of each skeletal cell type. By default, the original TAI formula considers the read count of each gene as a weight factor to give more substantial weight to highly expressed genes as they contribute more to cell type’s identity (Domazet-Loso & Tautz, 2010a). Beyond that, we also computed TAI values using only specifically expressed genes, which may refine the obtained patterns. To identify the specifically expressed genes, we performed differential gene expression analysis using Seurat’s *FindAllMarkers* command, selecting upregulated genes that show a minimum 25% difference in the fraction of detection (min.diff.pct = 0.25) between the focal cell type and the rest of the cell populations, thereby ensuring that the selected genes are more specific to the focal cell type. Additionally, the analysis was repeated after the number of cells for each cell type was downsampled using Seurat’s *subset* function (*n* = 50, *n* = 100).

The TAI values of cell types were statistically compared using Kruskal–Wallis and pairwise Wilcoxon post hoc tests. *P*-values were adjusted for multiple comparisons using Benjamini and Hochberg (BH) (Benjamini & Hochberg, 1995).

Partial TAI in a cell was calculated following the equation:


$$\begin{array}{l} TAI_{ps\left(\;j\right)}=\frac{ps_{j}e_{1}}{\overset{n}{\underset{i=1}{\sum }}e_{i}}+\frac{ps_{j}e_{2}}{\overset{n}{\underset{i=1}{\sum }}e_{i}}+\ldots +\frac{ps_{j}e_{m}}{\overset{n}{\underset{i=1}{\sum }}e_{i}} \end{array}$$

where TAI_ps(j)_ represents partial TAI of phylostratum (ps) *j* in a cell*, e*_*i*_ denotes the normalized read count of gene i in a cell, n is the total number of expressed genes in a cell, and *m* is the total number of genes that belong to phylostratum (ps) *j*.

### Average and relative expression analysis

We calculated the average expression of genes for each ps and every individual cell using the normalized total counts multiplied by a scaling factor of 10^6^. The relative expression of genes for a given phylostratum and cell type was subsequently calculated by linearly transforming the average expression values of genes from each phylostratum and cell type using the formula (Domazet-Loso & Tautz, 2010a):


$$\begin{array}{l} RE{\left(n\right)}_{s}=\frac{\bar{f}-{\bar{f}}_{min}}{{\bar{f}}_{max}-{\bar{f}}_{min}} \end{array}$$

where $\bar{f}$ represents the average expression of genes from phylostratum n for cell type s, and ${\bar{f}}_{min}$ and ${\bar{f}}_{min}$ denote the minimum and maximum mean expression values from phylostratum n across all cell types, respectively.

### Enrichment analysis

The enrichment of individual cell types across phylostrata was performed using the two-tailed hypergeometric test by comparing the distribution of all DEGs for each cell type (test set) across phylostrata with the overall distribution of DEGs from cell types of mesenchymal lineage (background set). Deviations in distributions are presented with log-odds, where log-odds of zero denote that the observed frequency of DEGs in a phylostrata equals the expected frequency from the background set. Enrichments in certain phylostrata indicate a significant number of genes are associated with the evolution of the cell type. The test was adjusted for multiple comparisons by correcting p-values using the BH procedure (Benjamini & Hochberg, 1995).

### GO enrichment

GO enrichment analysis of gene sets was performed and visualized with clusterProfiler R package and the *enrichGO* function correcting for multiple comparisons using the BH procedure (Benjamini & Hochberg, 1995). We used genome-wide *M. musculus* and *D. rerio* annotations, provided by the org.Mm.e.g.db (Carlson, 2019b) and org.Dr.e.g.db (Carlson, 2019a) R packages, respectively, as a background set.

## Supplementary Material

qraf012_suppl_Supplementary_Figures_S1-S9

qraf012_suppl_Supplementary_Tables_S1-S18

## Data Availability

Mouse single-cell dataset is available at NCBI (Geo accession number GSE142425). Zebrafish single-cell datasets are available at FaceBase (Record ID 5-DAQ4) and Zebrahub (https://zebrahub.ds.czbiohub.org). Phylostratigraphy data are presented in the supplementary information. The code used in this study is available on GitLab at [https://gitlab.gwdg.de/damatac/Evolutionary-trends-in-the-emergence-of-skeletal-cell-types].

## References

[CIT0001] Abzhanov, A., Rodda, S. J., McMahon, A. P., & Tabin, C. J. (2007). Regulation of skeletogenic differentiation in cranial dermal bone. Development, 134(17), 3133–3144. https://doi.org/10.1242/dev.002709 17670790

[CIT0002] Aghajanian, P., & Mohan, S. (2018). The art of building bone: Emerging role of chondrocyte-to-osteoblast transdifferentiation in endochondral ossification. Bone Research, 6, 19. https://doi.org/10.1038/s41413-018-0021-z 29928541 PMC6002476

[CIT0003] Altschul, S. F., Gish, W., Miller, W., Myers, E. W., & Lipman, D. J. (1990). Basic local alignment search tool. Journal of Molecular Biology, 215(3), 403–410. https://doi.org/10.1016/S0022-2836(05)80360-2 2231712

[CIT0004] Alvarez, J., & Serra, R. (2004). Unique and redundant roles of Smad3 in TGF-β-mediated regulation of long bone development in organ culture. Developmental Dynamics, 230(4), 685–699. https://doi.org/10.1002/dvdy.20100 15254903

[CIT0005] Anari, S. S., de Ridder, D., Schranz, M. E., & Smit, S. (2018). Efficient inference of homologs in large eukaryotic pan-proteomes. BMC Bioinformatics, 19. 10.1186/s12859-018-2362-4 PMC6158922 30257640

[CIT0006] Arendt, D. (2005). Genes and homology in nervous system evolution: Comparing gene functions, expression patterns, and cell type molecular fingerprints. Theory in Biosciences, 124(2), 185–197. https://doi.org/10.1007/BF02814483 17046355

[CIT0007] Arendt, D. (2008). The evolution of cell types in animals: Emerging principles from molecular studies. Nature Reviews Genetics, 9(11), 868–882. https://doi.org/10.1038/nrg2416 18927580

[CIT0008] Arendt, D. (2020). The evolutionary assembly of neuronal machinery. Current Biology, 30(10), R603–R616. https://doi.org/10.1016/j.cub.2020.04.008 32428501

[CIT0009] Arendt, D., Musser, J. M., Baker, C. V. H., Bergman, A., Cepko, C., Erwin, D. H., Pavlicev, M., Schlosser, G., Widder, S., Laubichler, M. D., & Wagner, G. P. (2016a). The origin and evolution of cell types. Nature Reviews Genetics, 17(12), 744–757. https://doi.org/10.1038/nrg.2016.127 27818507

[CIT0010] Arendt, D., Musser, J. M., Baker, C. V. H., Bergman, A., Cepko, C., Erwin, D. H., Pavlicev, M., Schlosser, G., Widder, S., Laubichler, M. D., & Wagner, G. P. (2016b). The origin and evolution of cell types. Nature Reviews Genetics, 17(12), 744–757. https://doi.org/10.1038/nrg.2016.127 27818507

[CIT0011] Arlt, A., & Schäfer, H. (2011). Role of the immediate early response 3 (IER3) gene in cellular stress response, inflammation and tumorigenesis. European Journal of Cell Biology, 90(6-7), 545–552. https://doi.org/10.1016/j.ejcb.2010.10.002 21112119

[CIT0012] Babonis, L. S., Enjolras, C., Ryan, J. F., & Martindale, M. Q. (2022). A novel regulatory gene promotes novel cell fate by suppressing ancestral fate in the sea anemone. Proceedings of the National Academy of Sciences of the United States of America, 119(19), e2113701119. https://doi.org/10.1073/pnas.2113701119 35500123 PMC9172639

[CIT0013] Barrera-Redondo, J., Lotharukpong, J. S., Drost, H. G., & Coelho, S. M. (2023). Uncovering gene-family founder events during major evolutionary transitions in animals, plants and fungi using GenEra. Genome Biology, 24(1), 54. https://doi.org/10.1186/s13059-023-02895-z 36964572 PMC10037820

[CIT0014] Barrionuevo, F., & Scherer, G. (2010). SOX E genes: SOX9 and SOX8 in mammalian testis development. International Journal of Biochemistry and Cell Biology, 42(3), 433–436. https://doi.org/10.1016/j.biocel.2009.07.015 19647095

[CIT0015] Beard, J. A., Tenga, A., & Chen, T. S. (2015). The interplay of NR4A receptors and the oncogene-tumor suppressor networks in cancer. Cellular Signalling, 27(2), 257–266. https://doi.org/10.1016/j.cellsig.2014.11.009 25446259 PMC4276441

[CIT0016] Benjamini, Y., & Hochberg, Y. (1995). Controlling the false discovery rate – A practical and powerful approach to multiple testing. Journal of the Royal Statistical Society Series B-Statistical Methodology, 57, 289–300.

[CIT0017] Bonucci, E., Silvestrini, G., & Bianco, P. (1992). Extracellular alkaline-phosphatase activity in mineralizing matrices of cartilage and bone – Ultrastructural-localization using a cerium-based method. Histochemistry, 97(4), 323–327. https://doi.org/10.1007/BF00270033 1618646

[CIT0018] Boonrungsiman, S., Gentleman, E., Carzaniga, R., Evans, N. D., McComb, D. W., Porter, A. E., & Stevens, M. M. (2012). The role of intracellular calcium phosphate in osteoblast-mediated bone apatite formation. Proceedings of the National Academy of Sciences of the United States of America, 109(35), 14170–14175. https://doi.org/10.1073/pnas.1208916109 22879397 PMC3435222

[CIT0019] Brazeau, M. D., Giles, S., Dearden, R. P., Jerve, A., Ariunchimeg, Y., Zorig, E., Sansom, R., Guillerme, T., & Castiello, M. (2020). Endochondral bone in an Early Devonian “placoderm” from Mongolia. Nature Ecology & Evolution, 4(11), 1477–1484. https://doi.org/10.1038/s41559-020-01290-2 32895518

[CIT0020] Brunet, T., & Arendt, D. (2016). Animal evolution: The hard problem of cartilage origins. Current Biology, 26(14), R685–R688. https://doi.org/10.1016/j.cub.2016.05.062 27458918

[CIT0021] Callier, V. (2020). Understanding the evolution of cell types to explain the roots of animal diversity. Proceedings of the National Academy of Sciences of the United States of America, 117(11), 5547–5549. https://doi.org/10.1073/pnas.2002403117 32184373 PMC7084151

[CIT0022] Carlson M. (2019a). *org.Dr.eg.db: Genome wide annotation for Zebrafish. Version R package version 3.8.2*.

[CIT0023] Carlson M. (2019b). *org.Mm.eg.db: Genome wide annotation for Mouse. Version R package version 3.8.2*.

[CIT0024] Carroll, S. B. (2008). Evo-devo and an expanding evolutionary synthesis: A genetic theory of morphological evolution. Cell, 134(1), 25–36. https://doi.org/10.1016/j.cell.2008.06.030 18614008

[CIT0025] Carter, A. M., & Mess, A. (2007). Evolution of the placenta in eutherian mammals. Placenta, 28(4), 259–262. https://doi.org/10.1016/j.placenta.2006.04.010 16780944

[CIT0026] Chen, H., Tan, X. N., Hu, S., Liu, R. Q., Peng, L. H., Li, Y. M., & Wu, P. (2021). Molecular mechanisms of chondrocyte proliferation and differentiation. Frontiers in Cell and Developmental Biology, 9, 664168. https://doi.org/10.3389/fcell.2021.664168 34124045 PMC8194090

[CIT0027] Cole, A. G., & Hall, B. K. (2004). The nature and significance of invertebrate cartilages revisited: Distribution and histology of cartilage and cartilage-like tissues within the Metazoa. Zoology, 107(4), 261–273. https://doi.org/10.1016/j.zool.2004.05.001 16351944

[CIT0028] Corak, N., Anniko, S., Daschkin-Steinborn, C., Krey, V., Koska, S., Futo, M., Siroki, T., Woichansky, I., Opasic, L., Kifer, D., Tušar, A., Maxeiner, H. -G., Domazet-Lošo, M., Nicolaus, C., & Domazet-Lošo, T. (2023). Pleomorphic variants of Borreliella (syn. Borrelia) burgdorferi express evolutionary distinct transcriptomes. International Journal of Molecular Sciences, 24(6), 5594. https://doi.org/10.3390/ijms24065594 36982667 PMC10057712

[CIT0029] D’Elia, G., Fabre, P. H., & Lessa, E. P. (2019). Rodent systematics in an age of discovery: Recent advances and prospects. Journal of Mammalogy, 100(3), 852–871. https://doi.org/10.1093/jmammal/gyy179

[CIT0030] Delsuc, F., Tsagkogeorga, G., Lartillot, N., & Philippe, H. (2008). Additional molecular support for the new chordate phylogeny. Genesis, 46(11), 592–604. https://doi.org/10.1002/dvg.20450 19003928

[CIT0031] Domazet-Loso, M., Siroki, T., Simicevic, K., & Domazet-Loso, T. (2024). Macroevolutionary dynamics of gene family gain and loss along multicellular eukaryotic lineages. Nature Communications, 15(1), 2663. https://doi.org/10.1038/s41467-024-47017-w PMC10966110 38531970

[CIT0032] Domazet-Loso, T., Brajkovic, J., & Tautz, D. (2007). A phylostratigraphy approach to uncover the genomic history of major adaptations in metazoan lineages. Trends in Genetics, 23(11), 533–539. https://doi.org/10.1016/j.tig.2007.08.014 18029048

[CIT0033] Domazet-Loso, T., & Tautz, D. (2010a). A phylogenetically based transcriptome age index mirrors ontogenetic divergence patterns. Nature, 468(7325), 815–818. https://doi.org/10.1038/nature09632 21150997

[CIT0034] Domazet-Loso, T., & Tautz, D. (2010b). Phylostratigraphic tracking of cancer genes suggests a link to the emergence of multicellularity in metazoa. BMC Biology, 8, 66. https://doi.org/10.1186/1741-7007-8-66 20492640 PMC2880965

[CIT0035] Donoghue, P. C., & Sansom, I. J. (2002). Origin and early evolution of vertebrate skeletonization. Microscopy Research and Technique, 59(5), 352–372. https://doi.org/10.1002/jemt.10217 12430166

[CIT0036] Donoghue, P. C., Sansom, I. J., & Downs, J. P. (2006). Early evolution of vertebrate skeletal tissues and cellular interactions, and the canalization of skeletal development. The Journal of Experimental Zoology B: Molecular and Developmental Evolution, 306(3), 278–294. https://doi.org/10.1002/jez.b.21090 16555304

[CIT0037] Drost, H. G., Gabel, A., Liu, J. L., Quint, M., & Grosse, I. (2018). myTAI: Evolutionary transcriptomics with R. Bioinformatics, 34(9), 1589–1590. https://doi.org/10.1093/bioinformatics/btx835 29309527 PMC5925770

[CIT0038] Dufour, H. D., Chettouh, Z., Deyts, C., de Rosa, R., Goridis, C., Joly, J. S., & Brunet, J. F. (2006). Precraniate origin of cranial motoneurons. Proceedings of the National Academy of Sciences of the United States of America, 103(23), 8727–8732. https://doi.org/10.1073/pnas.0600805103 16735475 PMC1482646

[CIT0039] Eames, B. F., Amores, A., Yan, Y. L., & Postlethwait, J. H. (2012). Evolution of the osteoblast: Skeletogenesis in gar and zebrafish. BMC Evolutionary Biology, 12, 27. https://doi.org/10.1186/1471-2148-12-27 22390748 PMC3314580

[CIT0040] Eames, B. F., de la Fuente, L., & Helms, J. A. (2003). Molecular ontogeny of the skeleton. Birth Defects Research. Part C, Embryo Today: Reviews, 69(2), 93–101. https://doi.org/10.1002/bdrc.10016 12955855

[CIT0041] Eames, B. F., Sharpe, P. T., & Helms, J. A. (2004). Hierarchy revealed in the specification of three skeletal fates by Sox9 and Runx2. Developmental Biology, 274(1), 188–200. https://doi.org/10.1016/j.ydbio.2004.07.006 15355797

[CIT0042] Fabian, P., Tseng, K. C., Thiruppathy, M., Arata, C., Chen, H. J., Smeeton, J., Nelson, N., & Crump, J. G. (2022). Lifelong single-cell profiling of cranial neural crest diversification in zebrafish. Nature Communications, 13(1), 13. https://doi.org/10.1038/s41467-021-27594-w PMC8748784 35013168

[CIT0043] Farnum, C. E., Lee, R., O’Hara, K., & Urban, J. P. (2002). Volume increase in growth plate chondrocytes during hypertrophy: The contribution of organic osmolytes. Bone, 30(4), 574–581. https://doi.org/10.1016/s8756-3282(01)00710-4 11934648

[CIT0044] Filipowska, J., Tomaszewski, K. A., Niedzwiedzki, L., Walocha, J., & Niedzwiedzki, T. (2017). The role of vasculature in bone development, regeneration and proper systemic functioning. Angiogenesis, 20, 291–302. 28194536 10.1007/s10456-017-9541-1 PMC5511612

[CIT0045] Fisher, S., & Franz-Odendaal, T. (2012a). Evolution of the bone gene regulatory network. Current Opinion in Genetics and Development, 22(4), 390–397. https://doi.org/10.1016/j.gde.2012.04.007 22663778

[CIT0046] Fisher, S., & Franz-Odendaal, T. (2012b). Evolution of the bone gene regulatory network. Current Opinion in Genetics & Development, 22(4), 390–397. https://doi.org/10.1016/j.gde.2012.04.007 22663778

[CIT0047] Foster, B. L., Ao, M., Salmon, C. R., Chavez, M. B., Kolli, T. N., Tran, A. B., Chu, E. Y., Kantovitz, K. R., Yadav, M., Narisawa, S., Millán, J. L., Nociti, F. H., & Somerman, M. J. (2018). Osteopontin regulates dentin and alveolar bone development and mineralization. Bone, 107, 196–207. https://doi.org/10.1016/j.bone.2017.12.004 29313816 PMC5803363

[CIT0048] Fulda, S. (2009). Tumor resistance to apoptosis. International Journal of Cancer, 124(3), 511–515. https://doi.org/10.1002/ijc.24064 19003982

[CIT0049] Futo, M., Opasic, L., Koska, S., Corak, N., Siroki, T., Ravikumar, V., Thorsell, A., Lenuzzi, M., Kifer, D., Domazet-Loso, M., Vlahoviček, K., Mijakovic, I., & Domazet-Lošo, T. (2021). Embryo-like features in developing *Bacillus subtilis* biofilms. Molecular Biology and Evolution, 38(1), 31–47. https://doi.org/10.1093/molbev/msaa217 32871001 PMC7783165

[CIT0050] Gans, C., & Northcutt, R. G. (1983). Neural crest and the origin of vertebrates: A new head. Science, 220(4594), 268–273. https://doi.org/10.1126/science.220.4594.268 17732898

[CIT0051] Giam, M., Huang, D. C. S., & Bouillet, P. (2008). BH3-only proteins and their roles in programmed cell death. Oncogene, 27(Suppl 1), S128–S136. https://doi.org/10.1038/onc.2009.50 19641498

[CIT0052] Gillis, J. A. (2019). The development and evolution of cartilage. In Reference module in life sciences. Elsevier. https://doi.org/10.1016/B978-0-12-809633-8.90770-2

[CIT0053] Giovannone, D., Paul, S., Schindler, S., Arata, C., Farmer, D. T., Patel, P., Smeeton, J., & Crump, J. G. (2019). Programmed conversion of hypertrophic chondrocytes into osteoblasts and marrow adipocytes within zebrafish bones. Elife, 8, e42736. https://doi.org/10.7554/eLife.42736 30785394 PMC6398980

[CIT0054] Gomez-Picos, P., & Eames, B. F. (2015). On the evolutionary relationship between chondrocytes and osteoblasts. Frontiers in Genetics, 6, 297. https://doi.org/10.3389/fgene.2015.00297 26442113 PMC4585068

[CIT0055] Gong, Y. Y., Xiong, L., Li, X. J., Su, L., & Xiao, H. P. (2021). A novel mutation of gene leading to increase ER stress and cell apoptosis is associated an autosomal dominant form of Wolfram syndrome type 1. BMC Endocrine Disorders, 21(1). 10.1186/s12902-021-00748-z PMC8059287 33879153

[CIT0056] Hafemeister, C., & Satija, R. (2019). Normalization and variance stabilization of single-cell RNA-seq data using regularized negative binomial regression. Genome Biology, 20(1), 296. https://doi.org/10.1186/s13059-019-1874-1 31870423 PMC6927181

[CIT0057] Hall, B. K. (1975). Evolutionary consequences of skeletal differentiation. American Zoologist, 15(2), 329–350. https://doi.org/10.1093/icb/15.2.329

[CIT0058] Han, Y., & Lefebvre, V. (2008). L-Sox5 and Sox6 drive expression of the aggrecan gene in cartilage by securing binding of Sox9 to a far-upstream enhancer. Molecular and Cellular Biology, 28(16), 4999–5013. https://doi.org/10.1128/MCB.00695-08 18559420 PMC2519711

[CIT0059] Harazono, Y., Kho, D. H., Balan, V., Nakajima, K., Zhang, T. P., Hogan, V., & Raz, A. (2014). Galectin-3 leads to attenuation of apoptosis through Bax heterodimerization in human thyroid carcinoma cells. Oncotarget, 5(20), 9992–10001. https://doi.org/10.18632/oncotarget.2486 25393982 PMC4259453

[CIT0060] Harvey, V. L., Keating, J. N., & Buckley, M. (2021). Phylogenetic analyses of ray-finned fishes (Actinopterygii) using collagen type I protein sequences. Royal Society Open Science, 8(8), 201955. https://doi.org/10.1098/rsos.201955 34430038 PMC8355665

[CIT0061] Hecht, J., Stricker, S., Wiecha, U., Stiege, A., Panopoulou, G., Podsiadlowski, L., Poustka, A. J., Dieterich, C., Ehrich, S., Suvorova, J., Mundlos, S., & Seitz, V. (2008). Evolution of a core gene network for skeletogenesis in chordates. PLoS Genetics, 4(3), e1000025. https://doi.org/10.1371/journal.pgen.1000025 18369444 PMC2265531

[CIT0062] Hemmrich, G., Khalturin, K., Boehm, A. M., Puchert, M., Anton-Erxleben, F., Wittlieb, J., Klostermeier, U. C., Rosenstiel, P., Oberg, H. H., Domazet-Loso, T., Sugimoto, T., Niwa, H., & Bosch, T. C. G. (2012). Molecular signatures of the three stem cell lineages in hydra and the emergence of stem cell function at the base of multicellularity. Molecular Biology and Evolution, 29(11), 3267–3280. https://doi.org/10.1093/molbev/mss134 22595987

[CIT0063] Hughes, L. C., Orti, G., Huang, Y., Sun, Y., Baldwin, C. C., Thompson, A. W., Arcila, D., Betancur-R, R., Li, C. H., Becker, L., Bellora, N., Zhao, X., Li, X., Wang, M., Fang, C., Xie, B., Zhou, Z., Huang, H., Chen, S., … Shi, Q. (2018). Comprehensive phylogeny of ray-finned fishes (Actinopterygii) based on transcriptomic and genomic data. Proceedings of the National Academy of Sciences of the United States of America, 115(24), 6249–6254. https://doi.org/10.1073/pnas.1719358115 29760103 PMC6004478

[CIT0123] Ichikawa-Shindo, Y., Sakurai, T., Kamiyoshi, A., Kawate, H., Iinuma, N., Yoshizawa, T., Koyama, T., Fukuchi, J., Iimuro, S., Moriyama, N., Kawakami, H., Murata, T., Kangawa, K., Nagai, R., & Shindo, T. (2008). The GPCR modulator protein RAMP2 is essential for angiogenesis and vascular integrity. Journal of Clinical Investigation, 118(1), 29–39. https://doi.org/10.1172/JCI33022 18097473 PMC2147670

[CIT0065] Jandzik, D., Garnett, A. T., Square, T. A., Cattell, M. V., Yu, J. K., & Medeiros, D. M. (2015). Evolution of the new vertebrate head by co-option of an ancient chordate skeletal tissue. Nature, 518(7540), 534–537. https://doi.org/10.1038/nature14000 25487155

[CIT0066] Jenike, A. E., Jenike, K. M., Peterson, K. J., Fromm, B., & Halushka, M. K. (2023). Direct observation of the evolution of cell-type-specific microRNA expression signatures supports the hematopoietic origin model of endothelial cells. Evolution and Development, 25(3), 226–239. https://doi.org/10.1111/ede.12438 37157156 PMC10302300

[CIT0067] Karsenty, G., Kronenberg, H. M., & Settembre, C. (2009). Genetic control of bone formation. Annual Review of Cell and Developmental Biology, 25, 629–648. https://doi.org/10.1146/annurev.cellbio.042308.113308 19575648

[CIT0068] Kelly, N. H., Huynh, N. P. T., & Guilak, F. (2020). Single cell RNA-sequencing reveals cellular heterogeneity and trajectories of lineage specification during murine embryonic limb development. Matrix Biology, 89, 1–10. https://doi.org/10.1016/j.matbio.2019.12.004 31874220 PMC7282974

[CIT0069] Kin, K., Chen, Z. H., Forbes, G., & Schaap, P. (2022). Evolution of a novel cell type in Dictyostelia required gene duplication of a cudA-like transcription factor. Current Biology, 32(2), 428–437.e4. https://doi.org/10.1016/j.cub.2021.11.047 34883046 PMC8808424

[CIT0070] Kin, K., Nnamani, M. C., Lynch, V. J., Michaelides, E., & Wagner, G. P. (2015). Cell-type phylogenetics and the origin of endometrial stromal cells. Cell Reports, 10(8), 1398–1409. https://doi.org/10.1016/j.celrep.2015.01.062 25732829

[CIT0071] Komori, T. (2005). Regulation of skeletal development by the Runx family of transcription factors. Journal of Cellular Biochemistry, 95(3), 445–453. https://doi.org/10.1002/jcb.20420 15786491

[CIT0072] Korsmeyer, S. J., Wei, M. C., Saito, M., Weller, S., Oh, K. J., & Schlesinger, P. H. (2000). Pro-apoptotic cascade activates BID, which oligomerizes BAK or BAX into pores that result in the release of cytochrome. Cell Death and Differentiation, 7, 1166–1173. 11175253 10.1038/sj.cdd.4400783

[CIT0073] Kronenberg, H. M. (2003). Developmental regulation of the growth plate. Nature, 423(6937), 332–336. https://doi.org/10.1038/nature01657 12748651

[CIT0074] Lange, M., Granados, A., Vijaykumar, S., Bragantini, J., Ancheta, S., Kim, Y. J., Santhosh, S., Borja, M., Kobayashi, H., McGeever, E., Solak, A. C., Yang, B., Zhao, X., Liu, Y., Detweiler, A. M., Paul, S., Theodoro, I., Mekonen, H., Charlton, C., … Royer, L. A. (2024). A multimodal zebrafish developmental atlas reveals the state-transition dynamics of late-vertebrate pluripotent axial progenitors. Cell, 187(23), 6742–6759.e17. https://doi.org/10.1016/j.cell.2024.09.047 39454574

[CIT0076] Lauri, A., Brunet, T., Handberg-Thorsager, M., Fischer, A. H., Simakov, O., Steinmetz, P. R., Tomer, R., Keller, P. J., & Arendt, D. (2014). Development of the annelid axochord: Insights into notochord evolution. Science, 345(6202), 1365–1368. https://doi.org/10.1126/science.1253396 25214631

[CIT0077] Le Pabic, P., Dranow, D. B., Hoyle, D. J., & Schilling, T. F. (2022). Zebrafish endochondral growth zones as they relate to human bone size, shape and disease. Frontiers in Endocrinology, 13, 1060187. https://doi.org/10.3389/fendo.2022.1060187 36561564 PMC9763315

[CIT0078] Londin, E., Loher, P., Telonis, A. G., Quann, K., Clark, P., Jing, Y., Hatzimichael, E., Kirino, Y., Honda, S., Lally, M., Ramratnam, B., Comstock, C. E. S., Knudsen, K. E., Gomella, L., Spaeth, G. L., Hark, L., Katz, L. J., Witkiewicz, A., Rostami, A., … Rigoutsos, I. (2015). Analysis of 13 cell types reveals evidence for the expression of numerous novel primate- and tissue-specific microRNAs. Proceedings of The National Academy Of Sciences Of The United States Of America, 112(10), E1106–E1115. https://doi.org/10.1073/pnas.1420955112 25713380 PMC4364231

[CIT0079] Ma, F., & Zheng, C. (2023). Transcriptome age of individual cell types in *Caenorhabditis elegans*. Proceedings of The National Academy Of Sciences Of The United States Of America, 120(9), e2216351120. https://doi.org/10.1073/pnas.2216351120 36812209 PMC9992843

[CIT0080] Mallatt, J., & Chen, J. Y. (2003). Fossil sister group of craniates: Predicted and found. Journal of Morphology, 258(1), 1–31. https://doi.org/10.1002/jmor.10081 12905532

[CIT0081] Meulemans, D., & Bronner-Fraser, M. (2007). Insights from amphioxus into the evolution of vertebrate cartilage. PLoS One, 2(8), e787. https://doi.org/10.1371/journal.pone.0000787 17726517 PMC1950077

[CIT0082] Neme, R., & Tautz, D. (2013). Phylogenetic patterns of emergence of new genes support a model of frequent de novo evolution. BMC Genomics, 14, 117. https://doi.org/10.1186/1471-2164-14-117 23433480 PMC3616865

[CIT0083] Nguyen, J. K. B., & Eames, B. F. (2020). Evolutionary repression of chondrogenic genes in the vertebrate osteoblast. FEBS Journal, 287(20), 4354–4361. https://doi.org/10.1111/febs.15228 31994313

[CIT0084] Nowakowski, T. J., Rani, N., Golkaram, M., Zhou, H. R., Alvarado, B., Huch, K., West, J. A., Leyrat, A., Pollen, A. A., Kriegstein, A. R., Petzold, L. R., & Kosik, K. S. (2018). Regulation of cell-type-specific transcriptomes by microRNA networks during human brain development. Nature Neuroscience, 21(12), 1784–1792. https://doi.org/10.1038/s41593-018-0265-3 30455455 PMC6312854

[CIT0085] Oltvai, Z. N., Milliman, C. L., & Korsmeyer, S. J. (1993). Bcl-2 heterodimerizes in-vivo with a conserved homolog, bax, that accelerates programmed cell-death. Cell, 74(4), 609–619. https://doi.org/10.1016/0092-8674(93)90509-o 8358790

[CIT0086] Opferman, J. T., & Kothari, A. (2018). Anti-apoptotic BCL-2 family members in development. Cell Death and Differentiation, 25(1), 37–45. https://doi.org/10.1038/cdd.2017.170 29099482 PMC5729530

[CIT0087] Person, P., & Philpott, D. E. (1969). The nature and significance of invertebrate cartilages. Biological Reviews of the Cambridge Philosophical Society, 44(1), 1–16. https://doi.org/10.1111/j.1469-185x.1969.tb00819.x 4388837

[CIT0124] Qin, X., Jiang, Q., Nagano, K., Moriishi, T., Miyazaki, T., Komori, H., Ito, K., Mark, K. V. D., Sakane, C., Kaneko, H., & Komori, T. (2020). Runx2 is essential for the transdifferentiation of chondrocytes into osteoblasts. PLOS Genetics, 16(11), e1009169. https://doi.org/10.1371/journal.pgen.1009169 33253203 PMC7728394

[CIT0089] Quint, M., Drost, H. G., Gabel, A., Ullrich, K. K., Bönn, M., & Grosse, I. (2012). A transcriptomic hourglass in plant embryogenesis. Nature, 490(7418), 98–101. https://doi.org/10.1038/nature11394 22951968

[CIT0090] Roach, H. I., Aigner, T., & Kouri, J. B. (2004). Chondroptosis: A variant of apoptotic cell death in chondrocytes? Apoptosis, 9(3), 265–277. https://doi.org/10.1023/b:appt.0000025803.17498.26 15258458

[CIT0091] Rossier, V., Vesztrocy, A. W., Robinson-Rechavi, M., & Dessimoz, C. (2021). OMAmer: Tree-driven and alignment-free protein assignment to subfamilies outperforms closest sequence approaches. Bioinformatics, 37, 2866–2873. 33787851 10.1093/bioinformatics/btab219 PMC8479680

[CIT0092] Rychel, A. L., Smith, S. E., Shimamoto, H. T., & Swalla, B. J. (2006). Evolution and development of the chordates: Collagen and pharyngeal cartilage. Molecular Biology and Evolution, 23(3), 541–549. https://doi.org/10.1093/molbev/msj055 16280542

[CIT0093] Ryll, B., Sanchez, S., Haitina, T., Tafforeau, P., & Ahlberg, P. E. (2014). The genome of Callorhinchus and the fossil record: A new perspective on SCPP gene evolution in gnathostomes. Evolution and Development, 16(3), 123–124. https://doi.org/10.1111/ede.12071 24712871 PMC4238839

[CIT0094] Sasseville, A. M. J., Caron, A. W., Bourget, L., Klein, A. F., Dicaire, M. J., Rouleau, G. A., Massie, B., Langelier, Y., & Brais, B. (2006). The dynamism of PABPN1 nuclear inclusions during the cell cycle. Neurobiology of Disease, 23, 621–629. 16860991 10.1016/j.nbd.2006.05.015

[CIT0095] Sestak, M. S., Bozicevic, V., Bakaric, R., Dunjko, V., & Domazet-Loso, T. (2013). Phylostratigraphic profiles reveal a deep evolutionary history of the vertebrate head sensory systems. Frontiers in Zoology, 10(1), 18. https://doi.org/10.1186/1742-9994-10-18 23587066 PMC3636138

[CIT0096] Sestak, M. S., & Domazet-Loso, T. (2015). Phylostratigraphic profiles in zebrafish uncover chordate origins of the vertebrate brain. Molecular Biology and Evolution, 32(2), 299–312. https://doi.org/10.1093/molbev/msu319 25415965 PMC4298178

[CIT0097] Sivaraj, K. K., & Adams, R. H. (2016). Blood vessel formation and function in bone. Development, 143(15), 2706–2715. https://doi.org/10.1242/dev.136861 27486231

[CIT0098] Somekawa, S., Imagawa, K., Hayashi, H., Sakabe, M., Ioka, T., Sato, G. E., Inada, K., Iwamoto, T., Mori, T., Uemura, S., Nakagawa, O., & Saito, Y. (2012). Tmem100, an ALK1 receptor signaling-dependent gene essential for arterial endothelium differentiation and vascular morphogenesis. Proceedings of the National Academy of Sciences of the United States of America, 109(30), 12064–12069. https://doi.org/10.1073/pnas.1207210109 22783020 PMC3409742

[CIT0099] Steppan, S. J., & Schenk, J. J. (2017). Muroid rodent phylogenetics: 900-species tree reveals increasing diversification rates. PLoS One, 12(8), e0183070. https://doi.org/10.1371/journal.pone.0183070 28813483 PMC5559066

[CIT0100] Stuart, T., Butler, A., Hoffman, P., Hafemeister, C., Papalexi, E., Mauck, W. M., Hao, Y. H., Stoeckius, M., Smibert, P., & Satija, R. (2019). Comprehensive integration of single-cell data. Cell, 177(7), 1888–1902.e21. https://doi.org/10.1016/j.cell.2019.05.031 31178118 PMC6687398

[CIT0101] Supruniuk, K., & Radziejewska, I. (2021). MUC1 is an oncoprotein with a significant role in apoptosis (Review). International Journal of Oncology, 59(3), 68. https://doi.org/10.3892/ijo.2021.5248 34278474 PMC8360618

[CIT0102] Suzuki, T., Sakai, D., Osumi, N., Wada, H., & Wakamatsu, Y. (2006). Sox genes regulate type 2 collagen expression in avian neural crest cells. Development Growth and Differentiation, 48(8), 477–486. https://doi.org/10.1111/j.1440-169X.2006.00886.x 17026712

[CIT0103] Swanson, M. T., Oliveros, C. H., & Esselstyn, J. A. (2019). A phylogenomic rodent tree reveals the repeated evolution of masseter architectures. Proceedings of the Royal Society B-Biological Sciences, 286(1902), 20190672. https://doi.org/10.1098/rspb.2019.0672 PMC6532498 31064307

[CIT0104] Tanabe, Y., William, C., & Jessell, T. M. (1998). Specification of motor neuron identity by the MNR2 homeodomain protein. Cell, 95(1), 67–80. https://doi.org/10.1016/s0092-8674(00)81783-3 9778248

[CIT0105] Tang, F. C., Barbacioru, C., Wang, Y. Z., Nordman, E., Lee, C., Xu, N. L., Wang, X. H., Bodeau, J., Tuch, B. B., Siddiqui, A., Lao, K., & Surani, M. A. (2009). mRNA-Seq whole-transcriptome analysis of a single cell. Nature Methods, 6(5), 377–382. https://doi.org/10.1038/nmeth.1315 19349980

[CIT0106] Tarashansky, A. J., Musser, J. M., Khariton, M., Li, P., Arendt, D., Quake, S. R., & Wang, B. (2021). Mapping single-cell atlases throughout Metazoa unravels cell type evolution. Elife, 10, e66747. https://doi.org/10.7554/eLife.66747 33944782 PMC8139856

[CIT0107] Tarazona, O. A., Slota, L. A., Lopez, D. H., Zhang, G., & Cohn, M. J. (2016). The genetic program for cartilage development has deep homology within Bilateria. Nature, 533(7601), 86–89. https://doi.org/10.1038/nature17398 27111511

[CIT0108] Taylor, J. M., & Padykula, H. A. (1978). Marsupial trophoblast and mammalian evolution. Nature, 271(5645), 588–588. https://doi.org/10.1038/271588b0

[CIT0109] Torday, J. S., & Miller, W. B. (2018). Terminal addition in a cellular world. Progress in Biophysics & Molecular Biology, 135, 1–10. 29273444 10.1016/j.pbiomolbio.2017.12.003

[CIT0110] Upham, N. S., Esselstyn, J. A., & Jetz, W. (2019). Inferring the mammal tree: Species-level sets of phylogenies for questions in ecology, evolution, and conservation. PLoS Biology, 17(12), e3000494. https://doi.org/10.1371/journal.pbio.3000494 31800571 PMC6892540

[CIT0122] Vaškaninová, V. (2020). Bone of contention. Nature Ecology & Evolution, 4, 1447–1448. https://doi.org/10.1038/s41559-020-01300-3 32895517

[CIT0111] Wagner, G. P., Erkenbrack, E. M., & Love, A. C. (2019). Stress-induced evolutionary innovation: A mechanism for the origin of cell types. Bioessays, 41(4), e1800188. https://doi.org/10.1002/bies.201800188 30919472 PMC7202399

[CIT0112] Wagner, G. P., & Lynch, V. J. (2010). Evolutionary novelties. Current Biology, 20(2), R48–R52. https://doi.org/10.1016/j.cub.2009.11.010 20129035

[CIT0113] Wei, T. L., Zhang, N. N., Guo, Z. B., Chi, F., Song, Y., & Zhu, X. K. (2015). Wnt4 signaling is associated with the decrease of proliferation and increase of apoptosis during age-related thymic involution. Molecular Medicine Reports, 12(5), 7568–7576. https://doi.org/10.3892/mmr.2015.4343 26397044

[CIT0114] Wei, M. C., Zong, W. X., Cheng, E. H. Y., Lindsten, T., Panoutsakopoulou, V., Ross, A. J., Roth, K. A., MacCregor, G. R., Thompson, C. B., & Korsmeyer, S. J. (2001). Proapoptotic BAX and BAK: A requisite gateway to mitochondrial dysfunction and death. Science, 292, 727–730. 11326099 10.1126/science.1059108 PMC3049805

[CIT0115] Wilson, B. A., Foy, S. G., Neme, R., & Masel, J. (2017). Young genes are highly disordered as predicted by the preadaptation hypothesis of de novo gene birth. Nature Ecology and Evolution, 1(6), 0146–0146. https://doi.org/10.1038/s41559-017-0146 28642936 PMC5476217

[CIT0116] Woods, A., Wang, G., & Beier, F. (2007). Regulation of chondrocyte differentiation by the actin cytoskeleton and adhesive interactions. Journal of Cellular Physiology, 213(1), 1–8. https://doi.org/10.1002/jcp.21110 17492773

[CIT0117] Yang, L., Tsang, K. Y., Tang, H. C., Chan, D., & Cheah, K. S. E. (2014). Hypertrophic chondrocytes can become osteoblasts and osteocytes in endochondral bone formation. Proceedings of the National Academy of Sciences of the United States of America, 111(33), 12097–12102. https://doi.org/10.1073/pnas.1302703111 25092332 PMC4143064

[CIT0118] Zeng, H. K. (2022). What is a cell type and how to define it? Cell, 185(15), 2739–2755. https://doi.org/10.1016/j.cell.2022.06.031 35868277 PMC9342916

[CIT0119] Zhang, G. J., Miyamoto, M. M., & Cohn, M. J. (2006). Lamprey type II collagen and Sox9 reveal an ancient origin of the vertebrate collagenous skeleton. Proceedings of the National Academy of Sciences of the United States of America, 103, 3180–3185. 16492784 10.1073/pnas.0508313103 PMC1413883

[CIT0120] Zhu, M., Yu, X., Ahlberg, P. E., Choo, B., Lu, J., Qiao, T., Qu, Q., Zhao, W., Jia, L., Blom, H., & Zhu, Y.'an (2013). A Silurian placoderm with osteichthyan-like marginal jaw bones. Nature, 502(7470), 188–193. https://doi.org/10.1038/nature12617 24067611

[CIT0121] Zou, M., Guo, B. C., Tao, W. J., Arratia, G., & He, S. P. (2012). Integrating multi-origin expression data improves the resolution of deep phylogeny of ray-finned fish (Actinopterygii). Scientific Reports, 2, 665. https://doi.org/10.1038/srep00665 22993690 PMC3444750

